# Anti-*Staphylococcus aureus* potential of compounds from *Ganoderma* sp.: A comprehensive molecular docking and simulation approaches

**DOI:** 10.1016/j.heliyon.2024.e28118

**Published:** 2024-03-26

**Authors:** Trang Thi Thu Nguyen, Trinh Thi Tuyet Nguyen, Hoang Duc Nguyen, Tan Khanh Nguyen, Phu Tran Vinh Pham, Linh Thuy Thi Tran, Hong Khuyen Thi Pham, Phu Chi Hieu Truong, Linh Thuoc Tran, Manh Hung Tran

**Affiliations:** a Faculty of Biology and Biotechnology, University of Science, 227 Nguyen Van Cu, District 5, Ho Chi Minh City, 700000, Viet Nam; b Vietnam National University, Linh Trung, Thu Duc City, Ho Chi Minh City, 700000, Viet Nam; c Scientific Management Department, Dong A University, 33 Xo Viet Nghe Tinh, Hai Chau District, Da Nang City, 550000, Viet Nam; d VN-UK Institute for Research and Executive Education, The University of Danang, 158A Le Loi, Hai Chau District, Danang City, 550000, Viet Nam; e Faculty of Pharmacy, Hue University of Medicine and Pharmacy, Hue University, Hue, 530000, Viet Nam; f School of Medicine and Pharmacy, The University of Danang, Hoa Quy, Ngu Hanh Son District, Da Nang City, 550000, Viet Nam

**Keywords:** *Ganoderma*, Molecular docking simulation, Molecular dynamic, *Staphylococcus aureus*

## Abstract

In this study, a series of secondary metabolites from Ganoderma sp. were screened against *Staphylococcus aureus* protein targets, including as phosphotransacetylase, clumping factor A, and dihydrofolate reductase, using molecular docking simulations. The chemicals that showed the strongest binding energy with the targeted proteins were ganodermanontriol, lucidumol B, ganoderic acid J, ergosterol, ergosterol peroxide, 7-oxoganoderic acid Z, ganoderic acid AM1, ganosinoside A, ganoderic acid D, and 24*R*-ergosta-7,2*E*-diene-3*β*,5*α*,6*β*-triol. Interestingly, ganosinoside A showed the greatest affinity for the protein clumping factor A, a result validated by molecular dynamic simulation. Additionally, three natural *Ganoderma* sp. Strains as *Ganoderma lingzhi* VNKKK1903*, Ganoderma lingzhi* VNKK1905A2, and *Amauroderma subresinosum* VNKKK1904 were collected from Kon Ka Kinh National Park in central land of Vietnam and evaluated for their antibacterial activity against *Staphylococcus aureus* using an agar well diffusion technique. These results suggest that the fungal extracts and secondary metabolites may serve as valuable sources of antibiotics against *Staphylococcus aureus*. These findings provided an important scientific groundwork for further exploration of the antibacterial mechanisms of compounds derived from *Ganoderma* sp. in future research.

## 1 Rationale

*Staphylococci* (*Staphylococcus*) bacteria are common culprits behind human illnesses, often forming clusters resembling grape bunches and primarily inhabiting the skin and mucous membranes [[1], [2], [3]]. Amongst the *Staphylococcus* species, *Staphylococcus aureus* stands out due to its prevalence and ability to cause severe, potentially fatal conditions such as osteomyelitis, endocarditis, sepsis, meningitis, skin syndrome with scabs, toxic shock syndrome, and food poisoning [2,4,5]. *S. aureus* typically colonizes the skin and nasopharynx, posing a threat primarily to immunocompromised individuals [6]. Alarmingly, approximately 50% of current *S. aureus* infections are resistant to multiple antibiotics, including penicillin, methicillin, tetracycline, ampicillin, and erythromycin [1,3]. The overuse of antibiotics has led to the emergence of various resistant *S. aureus* strains, such as linezolid, quinupristin, and dalfopristin [1,7].

With the escalating challenge of antibiotic resistance, there is an urgent call to develop new, more potent antimicrobial agents as alternatives or adjuncts to current antibiotic therapies. Nature has long provided a rich source of medicinal compounds with antimicrobial properties, as various bacterial and fungal pathogens have developed resistance mechanisms. Mushrooms, in particular, contain a diverse range of bioactive compounds with unique chemical structures, rendering them valuable in the quest for novel antibacterial drugs. *Ganodermaceae* species (Polyporales) are medicinal mushrooms and have been integral to traditional medicine in China and other Asian countries for centuries. However, only recently has the antibacterial potential of these species gained attention in research. Among these, *Ganoderma lucidum* is currently under investigation for its potential therapeutic antibacterial properties [[8], [9], [10]].

In our previous investigation, we compiled a list of natural compounds sourced from *Ganoderma* fungal species within the Ganodermataceae family and subjected them to virtual screening within sortase A complexes via docking simulations [10]. Our findings revealed that among the active compounds, two triterpenoids, ganosinensin B and ganosinoside A, displayed the highest binding energies. Moreover, they exhibited consistent binding to *S. aureus* sortase A complexes over a 100 ns duration. To validate the computational outcomes, we procured two natural *Ganoderma* species, *G. multiplicatum* VNKKK1901 and *G. sinense* VNKKK1902, from Kon Ka Kinh National Park in central Vietnam and assessed their antibacterial efficacy against *S. aureus.* Our results underscore the potential antibacterial attributes of these extracts against *S. aureus.*

Kon Ka Kinh National Park (KKKNP) stands as a prominent natural reserve located at 14°9′22″–14°29′52″ N and 108°15′26″–108°27′25″ E, approximately 1000 m above sea level in the Central Highlands of Vietnam. KKKNP encompasses a diverse range of vegetation types, including mixed coniferous and broadleaf forests. The average temperature fluctuates between 21.8 °C and 23.6 °C, accompanied by a total rainfall ranging from 1466 to 2272 mm, fostering favorable conditions for the proliferation of Ganodermataceae fungal species. Nevertheless, research regarding *Ganoderma* species within KKKNP, encompassing their morphological, ecological, and antibacterial characteristics, remains limited.

In this study, we compiled a list of 80 secondary metabolites sourced from these fungi and conducted molecular docking against three protein targets: dihydrofolate reductase, clumping factor A, and phosphotransacetylase. To expand our anti-*S. aureus* screening program, we collected three additional natural fungi as *Ganoderma lingzhi* VNKKK1903, *Ganoderma lingzhi* VNKK1905A2, and *Amauroderma subresinosum* VNKKK1904, and tested their antimicrobial activity.

## 2 Procedure

### 2.1 Selection of secondary metabolites from Ganodermaceae

**Fig. 1:**
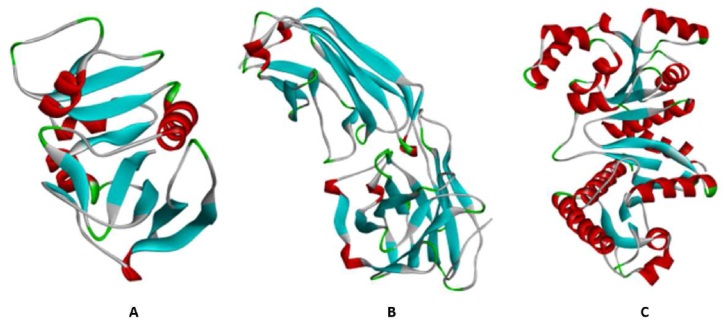
The 3D structure of (A) dihydrofolate reductase, (B) clumping factor A and (C) Phosphotransacetylase.

A serial of 80 natural compounds isolated from Ganodermaceae [[10], [11], [12], [13], [14], [15], [16]] was assembled for the molecular docking process, sourced from the database available at http://phytochem.nal.usda.gov (Supporting information Table S1). The 3D structures of these compounds were retrieved from PubChem (http://pubchem.ncbi.nlm.nih.gov) and prepared using MarvinSketch (ChemAxon, Cambridge, MA, USA). The co-crystallized ligand found within the respective binding site of proteins (Fig. 1) was utilized to validate the docking protocol. Subsequently, all ligands were converted to a dockable pdbqt format using Open Babel. The crystal ligand was successfully re-docked, exhibiting the same binding pose as the native conformation. Additionally, as a reference compound, the commercial antibiotic ciprofloxacin was docked to the three protein targets of *S. aureus*, namely dihydrofolate reductase (Fig. 1A), clumping factor A (Fig. 1B, and phosphotransacetylase (Fig. 1C).

### 2.2 Protein and ligand preparation

The crystal structures of dihydrofolate reductase (PDB ID: 2W9G, resolution: 1.95 Å), clumping factor A (PDB ID: 1N67, resolution: 1.90 Å), and phosphotransacetylase (PDB ID: 4E4R, resolution: 1.44 Å) were retrieved from the Research Collaboratory for Structural Bioinformatics Protein Data Bank (Fig. 1). To prepare these structures for molecular docking analysis, the existing ligands and water molecules were eliminated using Discovery Studio. Additionally, polar hydrogen atoms and Kollman charges were added to the proteins using Autodock tools (v. 1.5.6). Subsequently, the protein structures were converted to pdbqt format to facilitate the molecular docking process [7,17].

### 2.3 Molecular docking study

The chosen natural compounds were subjected to virtual screening on *S. aureus* proteins using AutoDock Vina. To facilitate the docking process, grid boxes were positioned to cover the active sites of the proteins, and these grid box coordinates were predicted using the CASTp server [18,19]. Docking scores, measured in kcal/mol, were obtained for each compound, and these scores were utilized to rank the compounds based on their binding affinity. Subsequently, the molecular interactions between the proteins and the selected ligands were visualized using Discovery Studio Visualizer. 3D and 2D interaction plots were generated to provide a comprehensive representation of the binding interactions between the proteins and ligands, offering insights into their complex interactions at a structural level.

### 2.4 Characterization and ADMET prediction

The ligands exhibiting the lowest binding energy towards the three studied proteins were selected for molecular characterization and ADMET prediction [20]. The SwissADME web tool was utilized to calculate the drug-likeness of the ligands based on Lipinski's rule of five, along with physicochemical properties such as the number of rotatable bonds (nRotB), total polar surface area (TPSA), molar refractivity (MR), and the logarithm of aqueous solubility (log S). Additionally, to predict the ligands' acute oral toxicity, the DL-AOT Prediction Server was employed.

### 2.5 Molecular dynamic simulation

The simulation protocol employed in this study followed our earlier methodology [20]. Molecular dynamics simulations were conducted using GROMACS 2020.4 to assess the stability of the complexes. The topology of the complexes was generated using the CHARMM36 force field, and the system was solvated in a truncated octahedral box filled with TIP3P water molecules through the CHARMM-GUI server [21]. Subsequently, a 100 ns molecular dynamics simulation was performed to analyze the dynamic behavior of the complexes.

### 2.6 Ganodermaceae sp. sample collection and extract preparation

The three natural basidiocarp mushrooms, namely *Ganoderma lingzhi* VNKKK1903, *Ganoderma* lingzhi VNKK1905A2, and *Amauroderma subresinosum* VNKKK1904, were collected in KKKNP, Vietnam (Table 1). Their identification specimens were conducted through morphological and phylogenetic methods using the 5.8S ITS rDNA gene. Following collection, the fruiting bodies were dried in a heating/drying oven (Memmert UFE 600, Buechenbach, Germany) at 50 °C until a constant weight was achieved. Subsequently, 100 g of pulverized basidiocarps was extracted using 1500 mL of 70% ethanol while being stirred at 120 rpm for 72 h. The solid and liquid components were separated using a fine-mesh strainer (0.2 cm), and the solids underwent another round of extraction with 1500 mL of 70% ethanol. The liquid extract was then filtered through Whatman No. 4 filter paper, concentrated under reduced pressure in a rotary evaporator (RV 10 digital V–C, IKA, Königswinter, Germany) at 40 °C until dryness, and finally dissolved in a 5% (v/v) dimethyl sulfoxide (DMSO) solution (Merck, Darmstadt, Germany) to achieve an initial concentration of 100 mg/mL. The extraction yield was determined using the ratio of dry fungal biomass before extraction to the dry extract weight after evaporation. Among the three strains, *G. lingzhi* VNKKK1903 exhibited the highest yield at 6.00 ± 0.02%, followed by *G. lingzhi* VNKKK1905A2 with 5.79 ± 0.16%, and *A. subresinosum* VNKKK1904 with 3.56 ± 0.21% (Table 1).

**Table 1:** Ganodermataceae specimens.

Specimens ID	Scientific name	Local name	The yield of extract (%)
VNKKK1903	*Ganoderma lingzhi*	Nấm lim xanh	6.00 ± 0.02
VNKKK1905A2	*Ganoderma lingzhi*	Nấm cổ cò	5.79 ± 0.16
VNKKK1904	*Amauroderma subresinosum*	Nấm hắc chi (nấm da trâu)	3.56 ± 0.21

### 2.7 Microorganisms and culture conditions

The bacterial strains utilized in this study comprised *S. aureus* ATCC 29213, along with four others: *Bacillus cereus* ATCC 11778, *Pseudomonas aeruginosa* ATCC 27853, *Escherichia coli* ATCC 35218, and *Klebsiella pneumoniae* ATCC 750603. These bacterial strains were sourced from the Institute of Public Health, Ho Chi Minh City, Vietnam. To prepare the bacterial cultures for experimentation, the bacteria were initially cultured on nutrient agar. Subsequently, they were transferred to Mueller-Hinton agar (MHA, Himedia, Mumbai, India) plates and incubated at 37 °C for 24 h to facilitate proper growth and development. The turbidity of the bacterial suspensions was evaluated using a spectrophotometer at a wavelength of 625 nm. The 0.5 McFarland turbidity standard served as a reference for assessing the turbidity level of the bacterial suspensions. The bacterial suspensions were then adjusted with sterile saline solution (0.85%) to achieve a final concentration of approximately 106 colony-forming units per milliliter (CFU/mL). This standardized concentration ensured consistency in the bacterial population employed in the experiments.

### 2.8 Disk diffusion method

Cell suspensions of *S. aureus* ATCC 29213, *Bacillus cereus* ATCC 11778, *Pseudomonas aeruginosa* ATCC 27853, *Escherichia coli* ATCC 35218, and *Klebsiella pneumoniae* ATCC 750603 were prepared at a concentration of 10^6^ CFU/mL. Subsequently, 1 mL of the prepared bacterial suspension was evenly spread over the surface of Mueller-Hinton agar (MHA) plates using sterile swabs to ensure uniform distribution of bacteria on the agar surface. Sterile filter paper discs with a diameter of 6.0 mm were impregnated with varying volumes of the tested extract to achieve different final amounts (1 mg, 2 mg, 3 mg, and 4 mg). Carefully, the impregnated filter paper discs were placed on top of the agar plates, ensuring even distribution across the surface. Ciprofloxacin, a known antibiotic with antibacterial properties, was used as the positive control on separate discs (5 μg/filter disk), while 5% dimethyl sulfoxide (DMSO) served as the negative control on separate discs. The prepared agar plates were initially incubated at 4 °C for 4 h to facilitate the diffusion of metabolites from the discs into the surrounding agar. Subsequently, the plates were further incubated at 35 ± 2 °C for 24 h to promote bacterial growth. After the incubation period, the diameter of the zone of bacterial growth inhibition around each disc (ZOI) was measured using a ruler. The ZOI indicated the area where bacterial growth was inhibited by the tested extract or controls [22].

### 2.9 Minimum inhibitory concentration (MIC) and minimum bactericidal concentration (MBC) determination

The Minimum Inhibitory Concentration (MIC) of the mushroom extract against microbial growth was determined using the broth microdilution method in 96-well microtiter plates [23,24]. Each well contained a total volume of 100 μL. The bacterial suspension was adjusted to a concentration of 1 × 10^6^ CFU/mL using saline solution. The mushroom extracts were dissolved in a 5% DMSO solution, while Mueller-Hinton broth (MHB) served as the growth medium for the bacteria. Subsequently, 5 × 10^5^ CFU of the bacterial inoculum was added to each well of the microtiter plate, and varying volumes of the mushroom extract were added to the wells to achieve a range of final concentrations (ranging from 0.05 to 100 mg/mL). The microtiter plates containing the bacteria and mushroom extract were then incubated at 35 ± 2 °C for 24 h, during which bacterial growth was monitored. The MIC value, representing the lowest concentration of the extract inhibiting microbial growth after 24 h of incubation, was determined using a colorimetric bacterial viability assay. This assay utilized a dye, 7-sodiooxy-3H-phenoxazin-3-one 10-oxide (resazurin), which changes color in the presence of viable bacteria. The microplate reader (BMG Labtech CLARIOstar®, Ortenberg, Germany) was employed to measure the color change and determine the Minimum Bactericidal Concentration (MBC). MBC was determined as the lowest concentration of the extract that completely eradicates bacterial growth. After incubation, 2.0 μL of the mixture from the well showing no visible growth was re-inoculated onto Mueller-Hinton broth. The absence of bacterial growth upon reinoculation indicated a 99.9% killing of the original inoculum. In this study, 5% DMSO served as the negative control, while ciprofloxacin was utilized as the positive control [25].

### 2.10 Statistical analysis

Quantitative data were obtained by triplicate experiments and analysis was performed with one-way ANOVA followed by Tukey Post Hoc test in Excel 2020.

## 3 Results

### 3.1 Interaction and binding affinity between compounds and the dihydrofolate reductase target

**Fig. 2:**
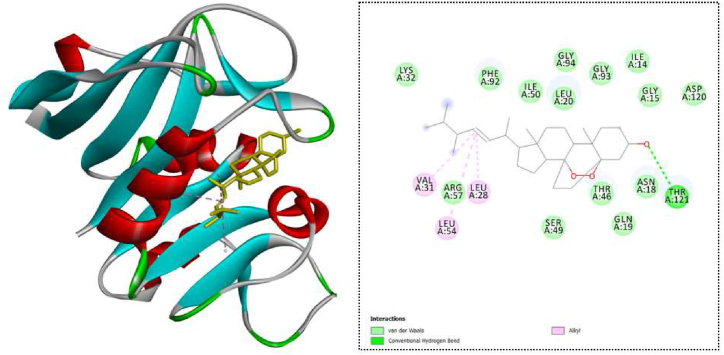
Ergosterol peroxide and dihydrofolate reductase interaction.

Among the selected compounds, four of them demonstrated promising results with docking scores greater than −9.5 kcal/mol, which is the docking score of ciprofloxacin. Ergosterol peroxide and ergosterol showed the highest binding energy in inhibiting the dihydrofolate reductase protein target (Table 2). For ergosterol peroxide, it interacted with dihydrofolate reductase through a hydrogen bond with THR121. Additionally, it engaged in hydrophobic interactions with VAL31, LEU28, and LEU54 (Fig. 2). As for ergosterol, it formed a hydrogen bond with ASN18 and also exhibited hydrophobic interactions with ILE50, ILE14, VAL31, LEU28, and PHE92. Another compound, 7-oxoganoderic acid Z, interacted with dihydrofolate reductase through two hydrogen bonds located at THR46 and THR96. Additionally, it engaged in hydrophobic interactions at THR46 and PHE92. Meanwhile, ganosinensin B demonstrated an even more extensive interaction profile. It formed six hydrogen bonds with ALA7, GLY93, THR46, ASN18, PRO21, and GLN19. Moreover, this compound participated in hydrophobic interactions with LEU20, ILE50, and LEU28 (additional information can be found in the supporting documentation).

**Table 2:** Docking results of top-ranked compounds in dihydrofolate reductase.

No	Compound	Docking score (kcal/mol)	Hydrogen bond	Hydrophobic interaction
1	Ergosterol peroxide	−10.2	THR121	VAL31, LEU28, LEU54
2	Ergosterol	−10.2	ASN18	ILE50, ILE14, VAL31, LEU28, PHE92
3	7- oxoganoderic acid Z	−10.2	THR46, THR96	THR46, PHE92
4	Ganosinensin B	−10.0	ALA7, GLY93, THR46, ASN18, PRO21, GLN19	LEU20, ILE50, LEU28

### 3.2 Interaction and binding affinity between compounds and clumping factor A

Among the selected compounds, we identified 47 compounds with docking scores greater than −8.1 kcal/mol, which is the docking score of ciprofloxacin (supporting information for the complete list). Two compounds, ganosinoside A and ganoderic acid AM1, exhibited the highest binding energy for inhibiting clumping factor A (Table 3).

**Table 3:** Docking results of top-ranked compounds on clumping factor A.

No	Compound	Docking score (kcal/mol)	Hydrogen bond	Hydrophobic interaction
1	Ganosinoside A	−10.4	ARG395, VAL288, ASN284, HIS252 THR397	PHE455, ILE488, AL490, TYR436, ASP385
2	Ganoderic acid AM1	−10.4	ILE339, PRO251, THR397, ARG395, PRO341	TYR436, PHE455, PRO452
3	Ganoderic acid J	−10.3		PRO251
4	Ergosterol peroxide	−10.2	THR397	TYR436, ILE448,PRO341,VAL288, VAL490, LYS343
5	Ganoderic acid D	−10.2	ILE339, TYR399, ARG395	
6	24R-ergosta-7,2E- diene-3β,5α,6β-triol	−10.1	THR397	TYR436, ILE488, LYS434, AL490

Ganosinoside A has formed five hydrogen bonds with clumping factor A at ARG395, VAL288, ASN284, HIS252, and THR397 and demonstrated hydrophobic interactions at PHE455, ILE488, AL490, TYR436, and ASP385. Ganoderic acid AM1 showed hydrogen bonding with ILE339, PRO251, THR397, ARG395, and PRO341, and exhibited hydrophobic interactions with TYR436, PHE455, and PRO452. Ganoderic acid J interacted through hydrophobic interactions with PRO251. Ergosterol peroxide developed one hydrogen bond at THR397, and had hydrophobic interactions at TYR436, ILE448, PRO341, VAL288, VAL490, and LYS343. About ganoderic acid D, this compound formed three hydrogen bonds at ILE339, TYR399, and ARG395. Meanwhile, 24*R*-ergosta-7,2*E*-diene-3*β*,5*α*,6*β*-triol interacted at THR397, TYR436, ILE488, LYS434, and AL490.

**Fig. 3:**
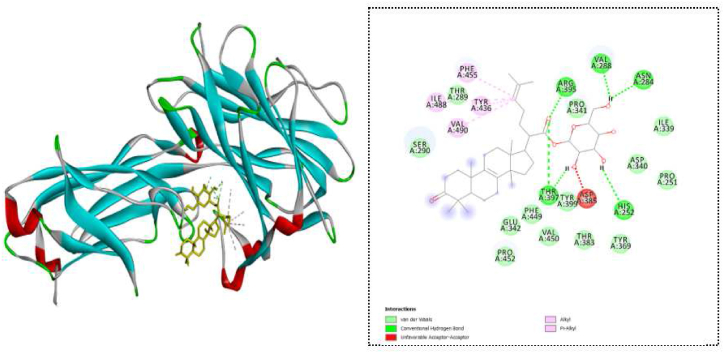
Ganosinoside A and clumping factor A interaction.

These detailed interactions between the compounds and clumping factor A provide valuable insights into their binding modes and potential mechanisms of action. The hydrogen bonding and hydrophobic interactions suggest that these compounds have a high affinity for clumping factor A, making them potential candidates for further investigation as antimicrobial agents or drug leads (Table 3 and Fig. 3).

### 3.3 Interaction with phosphotransacetylase

The results revealed 40 compounds with greater docking scores than ciprofloxacin, −7.1 kcal/mol (See supporting information). Notably, ganolucidic acid B showed the strongest binding energy in inhibiting phosphotransacetylase with docking score of −8.9 kcal/mol (Table 4).

**Table 4:** Docking results of top-ranked compounds on phosphotransacetylase.

No	Compound	Docking score (kcal/mol)	Hydrogen bond	Hydrophobic interaction
1	Ganolucidic acid B	−8.9	LEU299, LYS143, LYS187, GLN325, GLN297	LEU299
2	Ganoderenic acid D	−8.7	GLN325, LYS143, LYS187	LEU299
3	Ganoderic acid AM1	−8.6	SER191, LYS143, GLN325, GLY298, LEU299, LYS187, GLN297	
4	1-methoxy-2-(2-methoxyethenyl)-benzene	−8.3	SER191, GLN325, LYS232, GLU231	LEU299, GLY298
5	Jacareubin	−8.3	GLN325, LYS143, LYS187	LEU299

**Fig. 4:**
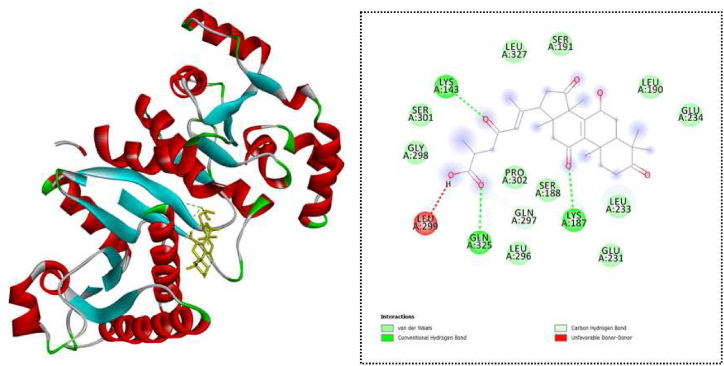
Ganolucidic acid B and phosphotransacetylase interaction.

Jacareubin (−8.3 kcal/mol) was in the top five compounds that formed three hydrogen bonds with phosphotransacetylase at GLN325, LYS143, and LYS187 and form one hydrophobic interaction at LEU299. Ganoderenic acid D also showed hydrogen bonding with GLN325, LYS143, and LYS187 and hydrophobic interaction with LEU299. Ganoderic acid AM1 formed seven hydrogen bonds at SER191, LYS143, GLN325, GLY298, LEU299, LYS187, and GLN297. 1-Methoxy-2-(2-methoxyethenyl)-benzene formed four hydrogen bonds at SER191, GLN325, LYS232, and GLU231 and interacted hydrophobically at LEU299 and GLY298 (Table 4). Meanwhile, ganolucidic acid B (−8.9 kcal/mol) interacted at LEU299, LYS143, LYS187, GLN325, and GLN297 by forming hydrogen bonds (Fig. 4).

### 3.4 Characterization and ADMET prediction

Among eighty studied compounds, six were chosen based on the criterion of lowest binding energy toward dihydrofolate reductase, clumping factor A and phosphotransacetylase proteins (top two compounds for each target). These ligands underwent screening for their drug-likeness using the SwissADME server, which adheres to Lipinski's rule of five. According to Lipinski's rule, a compound is considered drug-like if it meets the following criteria: molecular weight (MW) ≤ 500 Da, log of octanol/water partition coefficient (log P) < 5, number of hydrogen bond donors (nHBD) ≤ 5, and number of hydrogen bond acceptors (nHBA) ≤ 10. Additionally, other physicochemical properties such as number of rotatable bonds (nRotB), total polar surface area (TPSA), molar refractivity (MR), and log of aqueous solubility (log S) were predicted. These values serve as the basis for assessing intestinal absorption as well as oral bioavailability of drugs.

The results analyzed by SwissADME revealed that the six chosen compounds partially adhered to Lipinski's rule of five, as each compound violated only one criterion within this rule. Specifically, among these sterol derivatives, ergosterol peroxide and ergosterol exhibited a log P greater than 5, while the molecular weight of their counterparts exceeded 500 Da. However, the other physicochemical properties indicated good bioavailability and intestinal absorption, as the values of the number of rotatable bonds (nRotB) and total polar surface area (TPSA) did not exceed 10 and 140 (Å2), respectively (Table 5).

**Table 5:** Physicochemical properties of top-ranked compounds.

Compound	MW (g/mol)	Log P	nHBD	nHBA	TPSA (Å^2^)	MR	Lipinski Violation	Log S	nRotB
Ergosterol peroxide	428.65	5.77	1	3	38.69	128.08	1 (logP >5)	−6.46	4
Ergosterol	396.65	6.47	1	1	20.23	127.47	1 (log P > 5)	−6.72	4
Ganosinoside A	616.83	4.59	4	8	133.52	169.83	1 (MW > 500)	−6.51	8
Ganoderic acid AM1	514.65	3.41	2	7	125.81	140.09	1 (MW > 500)	−4.26	6
Ganolucidic acid B	502.68	4.06	3	6	111.90	140.85	1 (MW > 500)	−4.95	6
Ganoderenic acid D	512.63	3.29	2	7	125.81	139.61	1 (MW > 500)	−3.97	5

MW: molecular weight; log P: log of octanol/water partition coefficient; nHBD: number of hydrogen bond donor(s); nHBA: number of hydrogen bond acceptor(s); TPSA: total polar surface area; MR: molar refractivity; log S: log of solubility; nRotB: number of rotatable bond(s).

The ADME predictions were also generated by SwissADME and presented in Table 6. The indicator of skin permeability (log K_p_) of these compounds ranged from −7.96 to −3.44. Most of them were predicted to have low gastro-intestinal absorption. The results also pointed out that all six investigated compounds were not able to permeate the blood-brain barrier. A number of cytochrome-P play a vital role in drug biotransformation, including CYP1A2, CYP2C19, CYP2C9, CYP2D6, and CYP3A4. All six compounds were predicted not to inhibit CYP1A2, CYP2C19, and CYP2D6. Except for ergosterol peroxide and ergosterol, the remaining compounds were suggested to be a substrate of P-glycoprotein (P-gp). The binding energy and physicochemical property of these compounds indicate the potential of being used as therapeutic drugs when properly adjusted in dosage form. However, it would be important to investigate their toxicity for an accurate assessment of the potential harm these compounds may bring to the patient's health. In this study, acute oral toxicity of the chosen compounds was predicted by DL-AOT Prediction Server. All six compounds were classified as “Caution” group with the LD_50_ values ranged from 2.14 to 3.29 (mg/kg) (Table 7).

**Table 6:** ADME predictions of top-ranked compounds.

Compound	Log Kp (cm/s)	GI Abs	BBB per	Inhibitor Interaction					
				P-gp substrate	CYP1A2 Inhibitor	CYP2C19 Inhibitor	CYP2C9 Inhibitor	CYP2D6 Inhibitor	CYP3A4 Inhibitor
Ergosterol peroxide	−4.15	High	No	No	No	No	No	No	No
Ergosterol	−3.44	Low	No	No	No	No	Yes	No	No
Ganosinoside A	−6.26	Low	No	Yes	No	No	No	No	Yes
Ganoderic acid AM1	−7.61	Low	No	Yes	No	No	No	No	Yes
Ganolucidic acid B	−6.67	High	No	Yes	No	No	No	No	Yes
Ganoderenic acid D	−7.98	Low	No	Yes	No	No	No	No	No

Log K_p_: log of skin permeability; GI Abs: Gastro-intestinal absorption; BBB Per: Blood brain barrier permeability; P-gp: P-glycoprotein; CYP: cytochrome-P.

**Table 7:** Toxicity predicted by DL-AOT prediction server.

Compound	LD_50_ (mg/kg)	Toxicity
Ergosterol peroxide	2.15	Caution
Ergosterol	2.81	Caution
Ganosinoside A	3.29	Caution
Ganoderic acid AM1	2.31	Caution
Ganolucidic acid B	2.48	Caution
Ganoderenic acid D	2.14	Caution

### 3.5 Molecular dynamic simulation

**Fig. 5:**
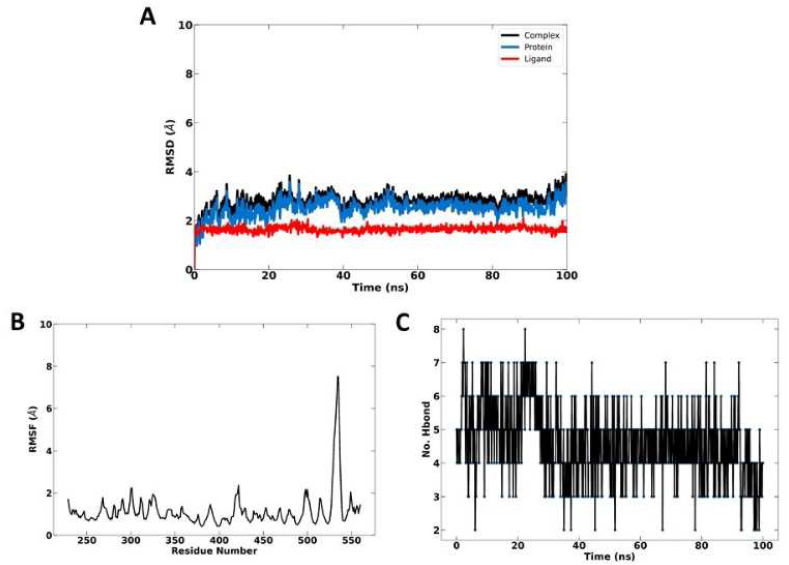
Dynamic simulation between ganosinoside A and clumping factor A for 100 ns. (A) RMSD. (B) RMSF. (C) Number of hydrogen bond.

Among the top compounds identified through docking, ganosinoside A exhibits the highest affinity for the clumping factor A protein. Consequently, we conducted molecular dynamics (MD) simulation experiments on this complex to establish a foundation for developing natural-origin compounds for the treatment of *S. aureus* infections (Fig. 5). We analyzed a 100 ns trajectory of the complex formed by the interaction of chemicals and proteins to evaluate the stability of both ligands and proteins. The simulation results indicate that the complex remained stable throughout the experiment's duration (Fig. 5A). The ligand contributes to the stability of specific interacting residues at the docking site, as evidenced by an analysis of the root mean square fluctuation (RMSF) (Fig. 5B). Furthermore, it was observed that the ligand consistently forms 4–6 hydrogen bonds with the protein, further enhancing the overall stability of the complex (Fig. 5C).

### 3.6 Antibacterial activity of extracts

In the subsequent investigation, three natural Ganodermaceae samples were collected, namely *Ganoderma lingzhi* VNKKK1903, *Ganoderma lingzhi* VNKK1905A2, and *Amauroderma subresinosum* VNKKK1904. Their extracts were subjected to *in vitro* testing to validate their inhibitory activity against bacterial strains. The antibacterial activities of the extracts are presented in Table 8, Table 9. All extracts (ranging from 1 to 4 mg/disk) exhibited zones of inhibition (ZOIs) against bacteria in a dose-dependent manner. Particularly noteworthy was the high activity observed in *G. lingzhi* VNKKK1903, which demonstrated significant antimicrobial ability against *S. aureus* starting from 2 mg/filter disk. This was followed by *G. lingzhi* VNKKK1903. Conversely, *G. lingzhi* VNKKK1905A2 and *A. subresinosum* VNKKK1904 exhibited weaker activity against *S. aureus*, with ZOIs of 6.33 ± 0.27 mm and 10.33 ± 0.54 mm, respectively (Table 8). The positive control antibiotic, ciprofloxacin, effectively inhibited the growth of *S. aureus* at 5 μg/filter disk, resulting in ZOIs above 30 mm.

**Table 8:** Diameter ZOI of different extracts of Ganodermataceae on the growth of *S. aureus* ATCC 29213.

Amount of dry extract per disc (mg)	ZOI (mm)^a^		
	*Ganoderma lingzhi* VNKKK1903	*Ganoderma lingzhi* VNKKK1905A2	*Amauroderma subresinosum* VNKKK1904
1	–	–	–
2	7.33 ± 0.27	–	–
3	7.67 ± 0.27	–	–
4	9.00 ± 0.47	6.33 ± 0.27	–
(+)	30.33 ± 0.27	31.67 ± 0.72	32.0 ± 0.0
(−)	–	–	–

a Data values are expressed as mean ± S.D. The analysis was conducted using one-way ANOVA (analysis of variance) followed by the Tukey post hoc test. (+) denotes the antibiotic positive control, ciprofloxacin (5 μg/filter disk), while (−) represents DMSO 5%.

**Table 9:** MIC and MBC of extracts on *S. aureus* ATCC 29213.

Extracts	Inhibitory parameters (mg/mL)^a^	
	MIC	MBC
VNKKK1903	9.3*	18.7**
VNKKK1905A2	6.2*	25.0**
VNKKK1904	3.1*	12.5**
CIP^b^	0.01	0.05

^a^Values are expressed as average of triplicated measures; Analysis was performed with one-way ANOVA analysis of variance followed by Tukey post hoc test; ^b^positive control, CIP: Ciprofloxacin (5 μg/filter disk); **P < 0.01; *P < 0.05.

The studied bacterial strains' varying susceptibilities to Ganodermataceae species and extract concentrations were also revealed by the MIC and MBC values (Table 9). With the lowest concentration MIC of 3.1 mg/mL, the extract of *A. subresinosum* VNKKK1904 was shown to be more potent than other extracts against *S. aureus*. *G. lingzhi* VNKK1905A2 followed with a MIC of 6.2 mg/mL. On the other hand, *G. lingzhi* VNKKK1903 extract had the highest MIC of 9.3 mg/mL. At 12.5 mg/mL for *S. aureus*, *A. subresinosum* VNKKK1904 showed the lowest dose of extract at which 99.9% of bacteria (5 × 105 CFU/mL) were destroyed (MBC), followed by G. lingzhi VNKKK1903. Ciprofloxacin was discovered to have a MIC of 0.01 mg/mL and an MBC of 0.05 mg/mL.

## 4 Discussion

Botanical antibiotics have garnered significant attention due to their potential to inhibit the survival of *S. aureus* bacteria. The Ganodermataceae family, in particular, has been extensively studied for its antimicrobial activities [26,27]. Notably, studies on *G. lucidum* have been conducted against *Bacillus subtilis, Salmonella typhimurium,* and *Escherichia coli* [[26], [27], [28]]. Furthermore, mixing *G. lucidum* extract with ampicillin, cefazolin, oxytetracycline, and chloramphenicol demonstrated encouraging outcomes against *Klebsiella oxytoca* and *B. subtilis* [[29], [30], [31]]. Research on *A. rugosum* in the genus Amauroderma showed that it was efficient against *Clostridium difficile, S. aureus, Pseudomonas aeruginosa,* and *E. coli* [32,33]. Remarkably, these triterpenoids exhibit higher susceptibility to gram-positive bacteria in contrast to gram-negative bacteria [34]. The two types of bacteria have different cell walls and membranes, which affect this sensitivity. The structure of gram-negative bacteria is more intricate, consisting of an outer membrane around peptidoglycan. By limiting the passage of molecules via its lipopolysaccharide layer and adding another layer of defense against dangerous substances, this outer membrane serves as an extra barrier. As a result, chemicals find it harder to enter gram-negative bacteria's guts and break through their outer membrane [35,36]. Gram-positive bacteria, on the other hand, have a simpler wall structure that is mainly made of peptidoglycan. It is nevertheless thick and hydrophilic. Because of this feature, chemicals can enter with less resistance, which makes it easier for them to absorb into the interior of gram-positive bacteria [35,36].

In this study, we initially curated several compounds classified as triterpenoids from the structure bank, previously identified in various Ganoderma species. These compounds were then employed to predict their interactions with three protein targets in *S. aureus*: dihydrofolate reductase, clumping factor A, and phosphotransacetylase. These specific proteins were selected due to their potential as targets in bacteria for compounds exhibiting antimicrobial activity against *S. aureus*. Through analyzing the interactions between these compounds and the chosen protein targets, our aim was to glean valuable insights into their potential as antimicrobial agents targeting *S. aureus*. Among the selected targets, dihydrofolate reductase (DHFR) is a folate-dependent enzyme present in *S. aureus* [37]. It plays a crucial role in catalyzing the biochemical reduction of dihydrofolate to tetrahydrofolate using NADPH. DHFR is involved in essential intracellular production pathways for purines like adenine and guanine, as well as other cellular components. As a result, DHFR becomes a potential target to address antimicrobial resistance. Specific inhibitors of DHFR can effectively block DNA replication in *S. aureus*, leading to bacterial death [37,38]. Another important target is phosphotransacetylase (PTA), also known as phosphate acetyltransferase and phosphoacylase. PTA is an enzyme comprising 328 amino acids and participates in distinct metabolic pathways [39]. It can cleave short-chain coenzyme A (CoA) esters, including acetyl-CoA, propionyl-CoA, and butyryl-CoA. *S. aureus* and other microbes utilize CoA esters via specific pathways to convert acetyl-CoA to acetate. Acetate serves as a growth substrate and is an end product of the metabolism of most microbes. Inhibiting PTA could effectively impede the growth and propagation of *S. aureus* [40]. Clumping factor A (ClfA) is the third target, a surface protein in *S. aureus* that plays a significant role as a major virulence factor. Its primary function is to bind to fibrinogen, a plasma protein, thereby inducing *S. aureus* to coagulate in plasma. This adhesion mechanism is crucial for the bacteria's ability to attach to host extracellular matrix proteins and is associated with infectious diseases [41]. Notably, vaccination with recombinant ClfA has demonstrated its protective potential against *S. aureus* infections. As a result, ClfA has been incorporated as an antigen in several multivalent *S. aureus* vaccines that are currently undergoing clinical trials [42,43]. These vaccines aim to leverage the immune response elicited by ClfA to provide protection against *S. aureus* infections, making ClfA an important target for vaccine development and potential therapeutic interventions. Regarding the dihydrofolate reductase target, among the selected compounds, 12 potential compounds exhibited docking scores greater than ciprofloxacin (−9.5 kcal/mol). Notably, ergosterol peroxide and ergosterol displayed the highest binding energy with docking scores of −10.4 kcal/mol, followed by 7-oxoganoderic acid Z and ganosinensin B (Table 2). Ergosterol peroxide interacted with the dihydrofolate reductase protein through a hydrogen bond with THR121 and formed hydrophobic bonds with VAL31, LEU28, and LEU54. Similarly, ergosterol exhibited a hydrogen bond interaction with ASN18 and five hydrophobic interactions with ILE50, ILE14, VAL31, LEU28, and PHE92.

Regarding clumping factor A, 47 compounds demonstrated docking scores greater than the docking score of ciprofloxacin. Among them, ganosinoside A and ganoderic acid AM1 exhibited the greatest binding energy for inhibiting clumping factor A, with docking scores of −10.4 kcal/mol. Notably, most of the top six compounds formed several hydrophobic interactions with the protein clumping factor A, except for ganoderic acid J, which had a hydrophobic interaction with PRO251 (Table 3 and Fig. 3). Concerning the phosphotransacetylase target, the results in Table 4 showed that 40 compounds exhibited greater docking scores than ciprofloxacin (−7.1 kcal/mol). Among these, ganolucidic acid B, a compound isolated from the fungus *Amauroderma amoiensis*, ranked among the top five compounds and demonstrated the strongest binding energy to phosphotransacetylase with a docking score of −8.9 kcal/mol.

Terpenoids are natural secondary metabolites with a cyclized hydrocarbon backbone, and they have been extensively studied for their wide range of biological functions. Numerous terpene compounds from *G. lucidum* and almost 400 triterpenoid compounds from reishi mushrooms have been discovered worldwide [44]. These terpenoids have demonstrated bacteriostatic and bactericidal properties, as well as their ability to inhibit oxidative phosphorylation and oxygen uptake in bacteria [35]. Terpenoids could influence both the polar and nonpolar transmembrane routes in bacterial cell membranes by interacting with the hydrophilic heads and hydrophobic tails of phospholipids [45]. Terpenoid classes may affect the bacterial efflux pumps that help remove harmful substances from bacterial cells, which could lead to a decrease in the resistance of bacteria to antibiotics [46]. Terpenoids could suppress virulence factors, which are essential for bacteria to cause disease and elude the immune system of the host [47]. Additionally, it has been shown that these substances prevent the development of bacterial biofilms, which are multicellular structures that protect bacteria from immune system agents and antimicrobial agents [[47], [48], [49], [50]]. Nevertheless, significant secondary structural changes can be induced by small molecules interacting with protein surfaces, and these alterations are essential for progressing the field of drug design. These alterations may affect the specificity and affinity with which small molecules attach to the target protein, which may improve the therapeutic effects of the molecules [17,18,47].

Two widely used methods, the agar plate diffusion method, and the broth microdilution method, serve distinct roles in evaluating the antimicrobial activity of compounds. The agar plate diffusion method assesses compound diffusion through agar, forming inhibition zones around discs to indicate antimicrobial activity. On the other hand, a more direct and quick interaction between substances and bacteria is made possible by the broth microdilution approach, which involves suspending compounds and bacteria in a liquid media [32,33].

One potential explanation for divergent results lies in the presence of polysaccharides in mushroom extracts [32,33]. Polysaccharides, often characterized by high molecular weights, may impede diffusion in agar, resulting in diminished or delayed antimicrobial zone formation [32,33,46]. However, the broth microdilution method, with compounds dispersed in a liquid medium, enables more effective interactions with bacterial membrane and cell wall structures, leading to heightened antimicrobial effects [32,33]. To gain a comprehensive understanding of the relationship between *Ganoderma* species compounds and their antimicrobial activity against *S. aureus*, further investigation and experimental validation are imperative. The validity and importance of research findings can be increased by including databases with data on the targets and composition of Ganoderma species as well as further clinical evidence. Adopting a multi-faceted approach, encompassing data mining, analysis, and experimental validation, is pivotal for advancing our comprehension of the potential therapeutic applications of *Ganoderma* compounds and their efficacy against *S. aureus*.

## 5 Conclusions

In conclusion, eighty natural compounds from *Ganoderma* species were gathered and put through a docking simulation-based virtual screening process. The findings indicated that the compounds with the strongest binding affinities to target proteins, such as dihydrofolate reductase (DHFR), clumping factor A (ClfA), and phosphotransacetylase (PTA), were ganodermanontriol, lucidumol B, ganoderic acid J, ergosterol, ergosterol peroxide, 7-oxoganoderic acid Z, ganoderic acid D, 24*R*-ergosta-7,2*E*-diene-3β, 5α, 6β-triol, and ganosinensin B. Molecular dynamic simulation has demonstrated that ganosinoside A has the highest affinity for the clumping factor A protein. Furthermore, the methanol extracts of three *Ganoderma* sp. collected in the KKKNP of Vietnam showed strong antibacterial effectiveness against *S. aureus*. The findings of this study emphasize the potential of these fungal extracts and their secondary metabolites as valuable sources of antimicrobial agents. While further investigations are necessary to elucidate the underlying mechanisms of the antibacterial activity of these fungal extracts, this research suggests that substances derived from fungal species hold promise for the development of new antibiotics against *S. aureus*. This work adds to the expanding knowledges about the possible antibacterial properties of naturally occurring fungi-derived chemicals. The compounds that were found to have robust binding with target proteins may provide insights into the creation of new medicines to treat *S. aureus* infections. To completely comprehend the antibacterial mechanisms and establish the usefulness of these natural fungal extracts as antimicrobial agents, additional thorough research is necessary.

## Data availability

The authors declare that the data supporting the findings of this study are available within the article. The raw/derived data supporting the findings of this study are available from the corresponding author at request.

## Funding

This work was funded by the 10.13039/501100015501PhD Scholarship Programme of the Vingroup Innovation Foundation (VINIF), Vingroup Big Data Institute (VINBIGDATA), code VINIF.2020.TS.68 (TTTN). This research is also funded by 10.13039/100015547Vietnam National University, Ho Chi Minh City (VNU-HCM) under grant number C2021-18-12.

## Ethics declarations

Not applicable.

## CRediT authorship contribution statement

**Trang Thi Thu Nguyen:** Writing – review & editing, Writing – original draft, Visualization, Validation, Investigation, Formal analysis, Data curation. **Trinh Thi Tuyet Nguyen:** Formal analysis, Data curation. **Hoang Duc Nguyen:** Methodology, Investigation, Formal analysis. **Tan Khanh Nguyen:** Software, Resources, Investigation, Formal analysis, Data curation. **Phu Tran Vinh Pham:** Resources, Methodology, Investigation, Formal analysis, Data curation. **Linh Thuy Thi Tran:** Software, Methodology, Investigation, Formal analysis. **Hong Khuyen Thi Pham:** Formal analysis, Data curation. **Phu Chi Hieu Truong:** Validation, Software, Resources, Formal analysis, Data curation, Conceptualization. **Linh Thuoc Tran:** Writing – review & editing, Writing – original draft, Validation, Supervision, Software, Resources, Methodology, Investigation, Conceptualization. **Manh Hung Tran:** Writing – review & editing, Writing – original draft, Supervision, Software, Resources, Project administration, Methodology, Investigation, Data curation, Conceptualization.

## Declaration of competing interest

The authors declare the following financial interests/personal relationships which may be considered as potential competing interests: Trang Thi Thu Nguyen reports financial support was provided by PhD Scholarship Programme of the Vingroup Innovation Foundation (VINIF), Vingroup Big Data Institute (VINBIGDATA), code VINIF.2020.TS.68 (TTTN). Trang Thi Thu Nguyen reports financial support was provided by Vietnam National University, Ho Chi Minh City (VNU-HCM) under grant number C2021-18-12.
