# Supplementary material for: Anti-Staphylococcus aureus potential of compounds from Ganoderma sp.: A comprehensive molecular docking and simulation approaches
Source: Heliyon. 2024 Mar 26;10(7):e28118. doi: 10.1016/j.heliyon.2024.e28118 (PMC11002548; doi:10.1016/j.heliyon.2024.e28118)
Supplement: Multimedia component 1 [file mmc1.docx]

**Supporting information to:**

**Anti-*Staphylococcus aureus* potential of compounds from *Ganoderma* sp.: A comprehensive molecular docking and simulation approaches**

Trang Thi Thu Nguyen^1,2^, Trinh Thi Tuyet Nguyen^1,2^, **Hoang Duc Nguyen^1,2^, Tan Khanh Nguyen^3^, Phu Tran Vinh Pham^4^,** Linh Thuy Thi Tran^5^, Hong Khuyen Thi Pham^6^, Phu Chi Hieu Truong^6^, **Linh Thuoc Tran**^1,2,*^, **Manh Hung Tran^6,*^**

^1^ Faculty of Biology and Biotechnology, University of Science, 227 Nguyen Van Cu, District 5, Ho Chi Minh city 700000, Vietnam.

^2^ Vietnam National University, Linh Trung, Thu Duc city, Ho Chi Minh city 700000, Vietnam.

^3^ Scientific Management Department, Dong A University, 33 Xo Viet Nghe Tinh, Hai Chau District, Da Nang city 550000, Vietnam.

^4^ VN-UK Institute for Research and Executive Education, The University of Danang, 158A Le Loi, Hai Chau District, Da Nang city 550000, Vietnam.

^5^ Faculty of Pharmacy, Hue University of Medicine and Pharmacy, Hue University, Hue 530000, Vietnam

^6^ School of Medicine and Pharmacy, The University of Danang, Hoa Quy, Ngu Hanh Son district, Da Nang city 550000, Vietnam.

**Corresponding author:** [tlthuoc@hcmus.edu.vn](mailto:tlthuoc@hcmus.edu.vn) (LT Tran); [tmhung@smp.udn.vn](mailto:tmhung@smp.udn.vn) (MH Tran)

**Abstract**

In this study, molecular docking simulation was applied to screen a list of eighty secondary metabolites from *Ganoderma* sp. on protein targets of *Staphylococcus aureus* including dihydrofolate reductase, clumping factor A, and phosphotransacetylase. Among the selected compounds, ganodermanontriol, lucidumol B, ganoderic acid J, ergosterol, ergosterol peroxide, 7-oxoganoderic acid Z, ganoderic acid AM1, ganosinoside A, ganoderic acid D, and 24*R*-ergosta-7,2*E*-diene-3*β,*5*α*, 6*β*-triol generated the most potent binding energy with the targeted proteins. Interestingly, ganosinoside A has the highest affinity for clumping factor A protein, which was confirmed by molecular dynamic simulation. In addition, three natural *Garnoderma* sp. as *Ganoderma lingzhi* VNKKK1903, *Ganoderma lingzhi* VNKK1905A2, and *Amauroderma subresinosum* VNKKK1904 were collected in Kon Ka Kinh National Park in Vietnam and test for their antibacterial activity against *S. aureus* using an agar well diffusion technique. These results propose that these fungal extracts and secondary metabolites might be valuable sources of antibiotics against *S. aureus*. These findings provided an important scientific basis for further research of the mechanism of anti-bacterial ofcompounds from *Ganoderma* sp. in the future.

**Keywords:** *Ganoderma*; molecular docking simulation; molecular dynamic; *Staphylococcus aureus*

**Table S1**. A list of compound from Ganoderma sp.

| **No** | **Name** |
| --- | --- |
| 1 | amaurosubresin |
| 2 | 5α,6α-epoxyergosta-8-ene-7-one-3β,23-diol |
| 3 | 24R-ergosta-7,22E-diene-3β-ol |
| 4 | 24R-ergosta-7,2E- diene-3β,5α,6β-triol |
| 5 | ergosterol peroxide |
| 6 | 24R-ergosta-5,22E-diene-3β,4β,15α,21- tetraol |
| 7 | 24R-5α,6α-epoxyergosta-8(14),22E-diene-15-one-3β,7α-diol |
| 8 | 24R-5α,6α-epoxyergosta-8,22E-diene-3β,7α-diol |
| 9 | jacareubin |
| 10 | 1-methoxy-2-(2-methoxyethenyl)-benzene |
| 11 | decanoic acid |
| 12 | pentadecanoic acid |
| 13 | cis-11-Octadecenoic acid |
| 14 | 9,12-octadecadienoic acid |
| 15 | ganoderic acid AM1 |
| 16 | lucidumol A |
| 17 | lucidumol B |
| 18 | ganodermanontriol |
| 19 | ganoderiol F |
| 20 | 7- oxoganoderic acid Z |
| 21 | ganopsoperic acid A |
| 22 | gannoderic acid B |
| 23 | gannoderic acid C1 |
| 24 | gannoderic acid E |
| 25 | ganodermanontriol |
| 26 | ganosporelactone A |
| 27 | ganosporelactone B |
| 28 | lucidumol C |
| 29 | ganoderic acid beta |
| 30 | lucidumol D |
| 31 | ganodermanondiol |
| 32 | ganoderiol F |
| 33 | ganoderic acid A |
| 34 | ganolucidic acid A |
| 35 | ganoderic acid γ |
| 36 | ganoderic acid δ |
| 37 | ganoderic acid ε |
| 38 | ganoderic acid ξ |
| 39 | ganoderic acid η |
| 40 | ganoderic acid θ |
| 41 | ganoderic acid C2 |
| 42 | ganoderic acid C6 |
| 43 | ganoderic acid G |
| 44 | ganoderic acid D |
| 45 | ganoderic acid H |
| 46 | methyl ganoderate A |
| 47 | methyl ganoderate B |
| 48 | 3-acetyl-lucidumol B |
| 49 | ergosterol palmitate |
| 50 | ergosterol |
| 51 | ergosterol peroxide |
| 52 | ganodermaside A |
| 53 | ganoderic acid G |
| 54 | ganodermadiol |
| 55 | ganoderic acid B |
| 56 | ganoderenic acid D |
| 57 | ganoderic acid AM1 |
| 58 | ganoderic acid J |
| 59 | ganoderiol A |
| 60 | ganodermatriol |
| 61 | ganodermanontriol |
| 62 | ganodine |
| 64 | ganosinensin B |
| 65 | ganosinensin C |
| 66 | ganosineniol A |
| 67 | ganosinoside A |
| 68 | ganoderic acid Jc |
| 69 | ganoderic acid Jd |
| 70 | ganodermatetraol |
| 71 | ganolucidate F |
| 72 | methyl lucidenate Ha |
| 73 | ganosinensine |
| 74 | ganoderiol E |
| 75 | ganoderiol F |
| 76 | ganoderma aldehyde |
| 77 | ganolucidic acid B |
| 78 | ganolucidic acid C |
| 79 | lucidadiol |
| 80 | ganosineniol B |
